# Revisiting the dynamic and thermodynamic processes driving the record-breaking January 2014 precipitation in the southern UK

**DOI:** 10.1038/s41598-019-39306-y

**Published:** 2019-02-27

**Authors:** Boutheina Oueslati, Pascal Yiou, Aglaé Jézéquel

**Affiliations:** Laboratoire des Sciences du Climat et de l’Environnement, UMR 8212 CEA-CNRS-UVSQ, IPSL and U Paris-Saclay, 91191 Gif-sur-Yvette Cedex, France

## Abstract

Many attribution studies of precipitation extreme events have attempted to estimate the thermodynamic contribution (linked to temperature changes) and the dynamic contribution (linked to the atmospheric circulation). Those studies are based on statistical decompositions of atmospheric fields, and essentially focus on the horizontal motion of the atmosphere. This paper proposes a framework that decomposes those terms from first physical principles, which include the vertical atmospheric motion that has often been overlooked. The goal is to take into account the driving processes of the extreme event. We revisit a recent example of extreme precipitation that was extensively investigated through its relation with the atmospheric circulation. We find that although the horizontal motion plays a minor (but important) role, the vertical motion yields a dominating contribution to the event that is larger than the thermodynamic contribution. This analysis quantifies the processes leading to high winter precipitation rates, and can be extended for further attribution studies.

## Introduction

**Figure 1:**
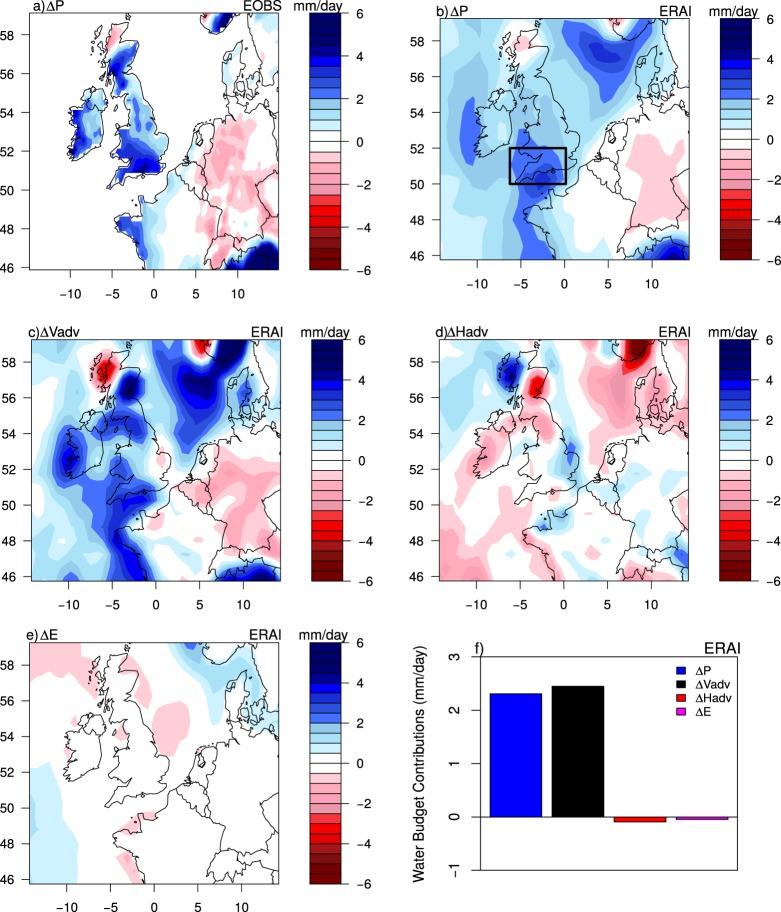
Monthly-mean anomalies for January 2014 of (**a**) EOBS^28^ precipitation, (**b**) ERA-I precipitation, (**c**) Vertical moisture advection, (**d**) Horizontal moisture advection, (**e**) Surface evaporation, (**f**) the four water budget contributions averaged over southern UK (50–52°N, 6.5°W–0°) as indicated by the black rectangle in panel (b)  over which spatial averages are computed using ERA-I. Anomalies are relative to 1981–2010 climatology.

During the 2013/14 winter, southern UK was affected by a spate of winter storms associated with a strengthening of the North Atlantic jet stream^1^. This exceptional situation resulted in heavy precipitation, with a record-breaking precipitation in southern UK (Fig. 1a)^1,2^ and north western France in January. Such extreme events are projected to intensify in this region as a response to planetary climate change^3,4^, with important impacts on societies. Understanding the driving processes of those events and their sensitivity to anthropogenic warming is, therefore, crucial to anticipate the future risks of flooding over the UK.

A fruitful approach in precipitation event attribution consists in separating dynamic and thermodynamic contributions^5–7^. The thermodynamic processes are associated with the enhancement of the atmospheric water vapor content, following the Clausius-Clapeyron equation^8–10^. They are robust across climate models and result in a nearly spatially homogeneous increase of precipitation^11^. The dynamic processes are related to the atmospheric circulation and remain highly uncertain at the regional scale^11–15^. They considerably influence the Clausius-Clapeyron scaling, strengthening for example, the daily heaviest precipitation^12–14,16^ and hourly precipitation extremes^17^. Therefore, considering the driving mechanisms separately is useful to deal with the highly uncertain dynamic changes and the robust thermodynamic changes in response to anthropogenic forcings.

Several studies attempted to quantify those individual contributions during the January 2014 heavy precipitation event. Schaller *et al*.^2^ and Vautard *et al*.^18^ concluded that a third of the increase in January precipitation can be attributed to changes in atmospheric dynamics and two thirds of the increase to thermodynamic changes. The two studies differ by the metric used to measure the effect of the circulation. Schaller *et al*.^2^ used the daily mean sea-level pressure (SLP) at a specific point as a proxy of the circulation. This metric is a poor description of the atmospheric dynamics and accounts for only one local feature of the flow. Vautard *et al*.^18^ applied a more general method based on flow analogues that are computed from monthly mean SLP over a regional domain (eastern north Atlantic ocean and Europe). However, this approach is sensitive to the way the similarity of the flows is approximated, either through weather regimes or flow analogues^18,19^. In addition, flows are characterized by mean SLP patterns that only describe the low-level horizontal atmospheric circulation. Such characterization misses the developing vertical circulation that controls the initiation and strength of convection. Therefore the statistical approaches that have been used might provide a partial view of the atmospheric circulation and estimate only a part of the dynamic contribution to extreme events. In particular, an explicit representation of the atmospheric vertical velocity in the available statistical diagnostics has been missing.

## Results

In this study, we propose an alternative framework to disentangle the dynamic and thermodynamic contributions. Changes in extreme precipitation are decomposed using a robust physical approach based on the atmospheric water budget (see Methods). This framework has been widely used in the tropics to relate local changes in precipitation to changes in atmospheric water vapor and circulation [e.g.^16,20,21^]. This method is applied to January 2014 precipitation to understand the physical drivers of this extreme event. It also provides a physically-based quantification of dynamic and thermodynamic contributions that might be useful for extreme event attribution. The analysis is carried out using the ERA-Interim (ERAI) reanalysis^22^, motivated by the horizontal resolution of this dataset (0.75°). The robustness of the results are tested using the NCEP reanalysis^23^ (Supplementary Material).

The monthly-mean pattern of precipitation anomaly during January 2014 is better represented by ERAI (Fig. 1b) compared to NCEP. This was also the case for daily variability. Both reanalyses, however, underestimate precipitation intensity. The monthly-mean water budget is computed to relate January 2014 precipitation anomalies to changes in the vertical moisture advection (${\rm{\Delta }}{V}_{adv}$), the horizontal moisture advection (${\rm{\Delta }}{H}_{adv}$) and surface evaporation (${\rm{\Delta }}E$) (Methods section and Fig. 1c–e).

January 2014 precipitation in southern UK is characterized by stronger than usual moisture vertical advection anomalies (larger than 2 mm/day on average for ERAI and NCEP) (Fig. 1c,f and Supplementary Fig. 1a). These positive anomalies moisten the troposphere by the vertical transport of moisture and sustain low-level moisture convergence. Abundant moisture in the atmospheric column and strong vertical motions resulted in heavy precipitation in southern UK. Horizontal moisture advection is small and negative at monthly time scale. Therefore, it contributes to drying the troposphere through the transport of dry air into rainy regions^24^ and reducing precipitation intensity (Fig. 1d,f). Surface evaporation is small over land and in particular, over southern UK (Fig. 1e,f). Overall, January 2014 precipitation is dominated by moisture convergence associated with vertical motion (Fig. 1c). The dominance of this physical mechanism in inducing heavy precipitation has already been highlighted in previous studies^11–13,15,16^ using climate models. In subtropical regions, changes in extreme ascent are explained by changes in the horizontal scale of ascending anomalies, which are associated with changes in vertical stability^15^.

**Figure 2:**
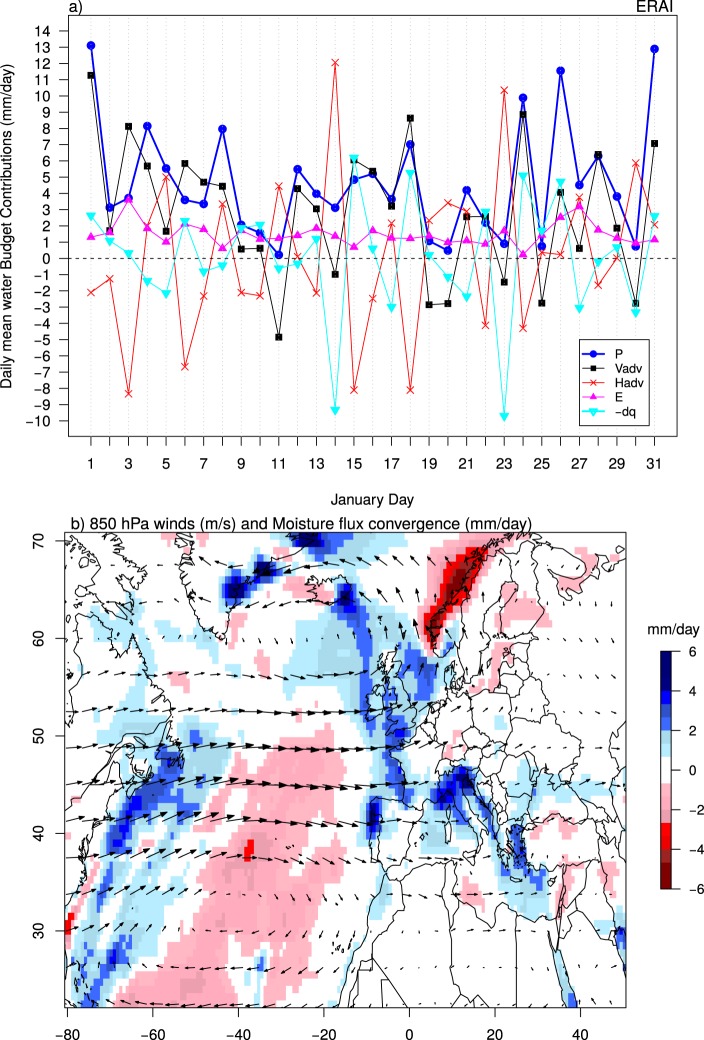
(**a**) Daily mean atmospheric water budget contributions for January 2014 averaged over southern UK (black rectangle in Fig. 1b), (**b**) Monthly-mean 850 hPa horizontal winds and vertically-integrated moisture flux convergence for January 2014. Positive (negative) values correspond to areas of moisture flux divergence (convergence).

At daily time-scale, vertical moisture advection is still the dominant process in generating intense precipitation (Fig. 2a), with a positive correlation of 0.8 between daily-mean $P$ and ${V}_{adv}$ in January 2014. Vertical advection moistens the troposphere through the vertical transport of moisture and is conducive to the development of convection at the same day of maximum vertical advection (Fig. 2a). This is the case for the heaviest rainy days of January 2014 (i.e. Jan. 1st, 4th, 18th, 24th and 31st), during which a ${V}_{adv}$ of 6 mm/day was needed to induce precipitation rates ranging between 6 to 13 mm/day. In contrast to the vertical moisture advection, horizontal moisture advection has, in most cases, an opposing effect on the heavy precipitation events (Fig. 2a). Positive horizontal moisture advection peaks 1 day (e.g. 15th, 24th, 31st of January) to several days (2 to 3 days) (e.g. 8th, 26th of January) before the maximum rainfall and becomes negative during and after the rainfall maximum (Fig. 2a). Thus it contributes to the moistening of the troposphere before the maximum precipitation and to its drying during the heavy rainfall events^24^.

Our analysis decomposes the sequence of events that led to a high accumulated precipitation. The horizontal advection ${H}_{adv}$ is a necessary precursor and the vertical advection ${V}_{adv}$ is necessary and sufficient once enough moisture is available (Fig. 2a).

To identify the origin of the low-level moistening through horizontal moisture advection, monthly-mean 850 hPa winds and the vertically-integrated moisture flux convergence are examined (Fig. 2b). Moisture convergence occurs over rainy regions, particularly over southern UK. Moisture divergence is localized over the subtropical and central North Atlantic, suggesting that this oceanic region is the primary source of moisture for the UK (Fig. 2b). Westerly winds over central North Atlantic were much stronger than normal during January 2014, favoured by a persistent zonal circulation^2^. These winds contributed to advect moisture eastward towards the UK causing heavy precipitation and flooding (Fig. 1a). Extra moisture might also have been transported from the subtropical North Atlantic by south-westerly winds, which is away from the divergence regions identified in Fig. 2b. This moisture results from larger oceanic evaporation in January 2014 associated with warmer oceans (Fig. 1e). January 2014 precipitation could therefore be connected to *atmospheric rivers*, which transport large flux of moisture from the subtropics to the mid-latitudes (Fig. 2b), leading to heavy precipitation and flooding over UK^25^. Back trajectory analyses are however needed to confirm the tropical origin of moisture during this event.

**Figure 3:**
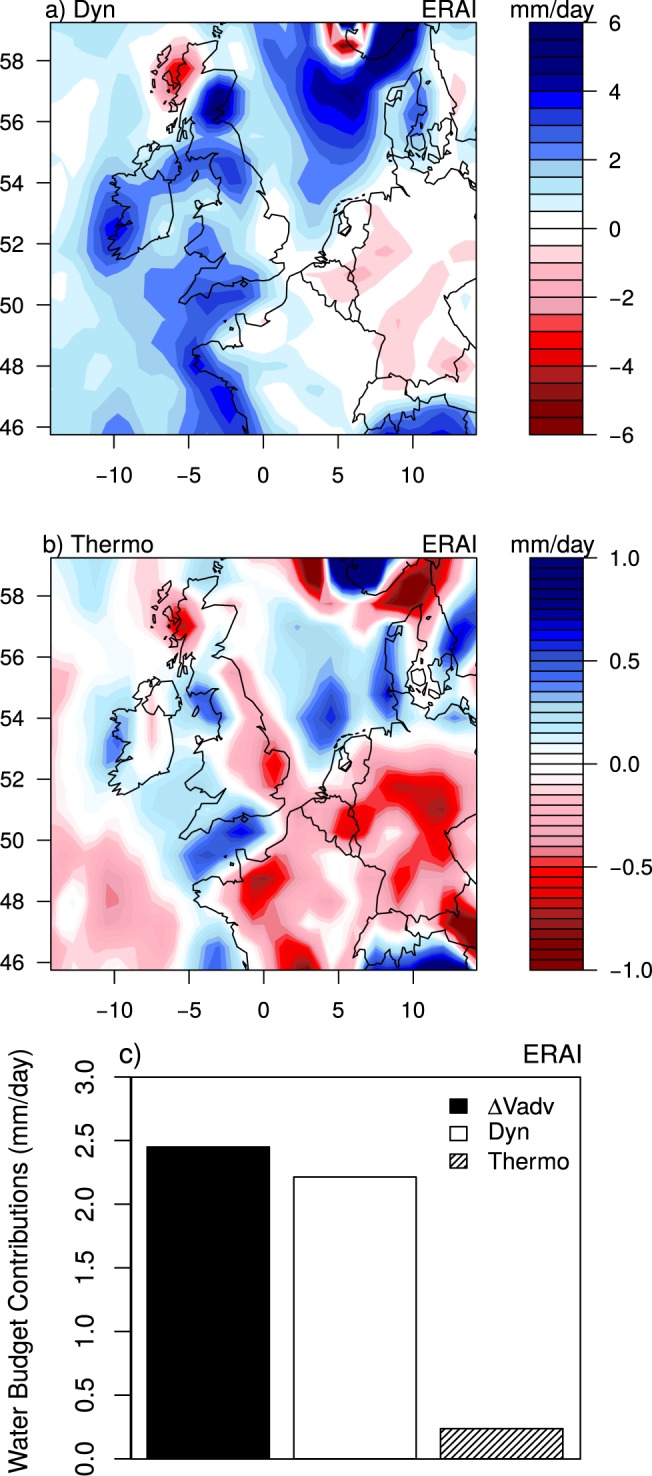
Monthly-mean anomalies of (**a**) dynamic and (**b**) thermodynamic contributions to precipitation anomaly during January 2014 derived from Eq. (3) using ERA-I, (**c**) As (**a**), but averaged over southern UK (black rectangle in Fig. 1b). Anomalies are relative to 1981–2010 climatology.

To further understand the mechanisms inducing heavy precipitation in southern UK, we focus on the dominant driver, i.e. the vertical moisture advection. ${\rm{\Delta }}{V}_{adv}$ is divided into thermodynamic and dynamic contributions (Methods section, Fig. 3 and Supplementary Fig. 1b). The thermodynamic component ($Thermo$) is associated with changes in water vapor that are largely dominated by the Clausius-Clapeyron relation^8,9^. The dynamic component ($Dyn$) is associated with changes in vertical velocity. $Dyn$ and $Thermo$ compute the vertically-integrated dynamic and thermodynamic changes and include the influence of temperature lapse-rate changes^26^. $Dyn$ is the main contributor to the vertical transport of moisture and contributes to more than 90% of ${\rm{\Delta }}{V}_{adv}$ over southern UK (Fig. 3a,c). $Thermo$ is very small (less than 1 mm/day in southern UK) and contributes only little to ${\rm{\Delta }}{V}_{adv}$ (Fig. 3b,c).

## Conclusions

The atmospheric circulation was a crucial element for January 2014 heavy precipitation. This extreme event was dynamically-induced by stronger vertical motions, which moistened the atmospheric column and promoted convection. Evaluating how anthropogenic climate change may alter the dynamic and thermodynamic contributions is essential to assess future projections of extreme precipitation. The $Dyn$ and $Thermo$ components are relevant metrics in that context. They yield a precise physical meaning at all vertical levels and at a regional scale. These metrics can be used in extreme event attribution studies (e.g.^2,18,19^) to provide a robust quantification of the role of the atmospheric circulation and water vapor in future changes in extreme precipitation. This approach can be applied consistently to reanalysis data or model simulations to analyze other wet winters. Our results do not necessarily contradict the existing event attribution papers: we find that the dominant factor for high precipitation is the vertical motion of the atmosphere. But long-term changes in this advection mechanism can be very small, compared to changes in the thermodynamic term in the extra-tropics. They can even be of opposite sign^11^. Evaluating those changes in a precise way is needed to gain confidence on the physical drivers of precipitation extremes. This can be done with our Eq. (3), from long model simulations or reanalyses. Those results follow the so-called storyline approach advocated by Shepherd^7^. This helps constraining potential changes of those components if a baseline climatology is altered.

This approach can be useful in assessing the risk of flooding over Europe at a seasonal time-scale. Indeed, understanding the processes responsible for heavy precipitation could highlight emerging forcings, e.g. particular states of sea-surface temperature, as precursors of heavy precipitation events.

## Methods

### Moisture budget

Starting from the vertically-integrated water budget at daily time-scale, regional precipitation (expressed in mm/day or equivalently in kg m^−2^ day^−1^) can be decomposed as:

1 $$\begin{array}{rcl}P & = & E-[\omega \cdot \frac{\partial q}{\partial p}]-[{\bf{V}}\cdot \nabla q]-[\frac{\partial q}{\partial t}]\\  & = & E+{V}_{adv}+{H}_{adv}-{\partial }_{t}q.\end{array}$$

In Eq. (1),  $E$ (mm/day) is surface (ocean or land) evaporation into the atmosphere, $\omega$ (Pa/day) is the vertical velocity, **V** (m/day) is the horizontal wind, $q$ (kg/kg) the specific humidity and $p$ (Pa or kg m^−1^ day^−2^) is the atmospheric pressure. Brackets denote a mass-weighted vertical integral from the surface ($p={p}_{s}$) to the top of the atmosphere ($p=0$). It is defined, for a quantity *A*, as $[A]={\int }_{0}^{{p}_{s}}\,A\frac{dp}{g}$, where $g$ is gravity acceleration (m day^−2^). ${V}_{adv}$, ${H}_{adv}$ and ${\partial }_{t}q$ represent respectively the vertical moisture advection, the horizontal moisture advection and the time derivative of $q$ (referred to as −*dq*).

${\partial }_{t}q$ corresponds to the change in atmospheric moisture storage. At monthly or longer time scales, this term is small compared to the other terms in the moisture budget equation and can be neglected^27^. The change in monthly-mean precipitation can therefore be expressed as:

2 $${\rm{\Delta }}P={\rm{\Delta }}E+{\rm{\Delta }}{V}_{adv}+{\rm{\Delta }}{H}_{adv}.$$

### Dynamic and thermodynamic contributions to precipitation changes

The vertical moisture advection is decomposed into a dynamic component ($Dyn$) related to vertical velocity changes and a thermodynamic component ($Thermo$) related to atmospheric water vapor changes. $Thermo$ provides a physically-based metric of the thermodynamic processes linked to Clausius-Clapeyron equation. Indeed, in a warming climate, as the atmosphere warms, saturation vapor pressure increases approximately following the Clausius-Clapeyron equation. It results in more moisture available in the atmosphere to condense into precipitation, favouring more intense rainy events. $Thermo$ is related to this mechanism and quantifies the contribution of moisture change in the enhancement of extreme precipitation.

3 $${\rm{\Delta }}{V}_{adv}=-\,[{\rm{\Delta }}\omega \cdot \overline{\frac{\partial q}{\partial p}}]-[\overline{\omega }\cdot {\rm{\Delta }}\frac{\partial q}{\partial p}]=Dyn+Thermo,$$

where the overbar indicates the 1981–2010 climatology mean.

## Data Availability

ERA-interim data are available from the ECMWF Public datasets web interface (http://apps.ecmwf.int/datasets). NCEP data are available from the NOAA Public datasets web interface (http://www.esrl.noaa.gov/psd/thredds/dodsC/Datasets).

## Supplementary file

Supplementary file
