# Supplementary material for: Revisiting the dynamic and thermodynamic processes driving the record-breaking January 2014 precipitation in the southern UK
Source: Sci Rep. 2019 Feb 27;9:2859. doi: 10.1038/s41598-019-39306-y (PMC6393453; doi:10.1038/s41598-019-39306-y)
Supplement: Supplementary file 1 — Supplementary file [file 41598_2019_39306_MOESM1_ESM.pdf]

Revisiting the dynamic and thermodynamic processes driving the record-breaking January 2014 precipitation in the southern UK

Supplementary Material

Boutheina Oueslati[1], Pascal Yiou[1], and Aglae Jezequel[1]

[1] *Laboratoire des Sciences du Climat et de l'Environnement, UMR CEA-CNRS-UVSQ, IPSL and U Paris-Saclay, 91191 Gif-sur-Yvette Cedex, France*

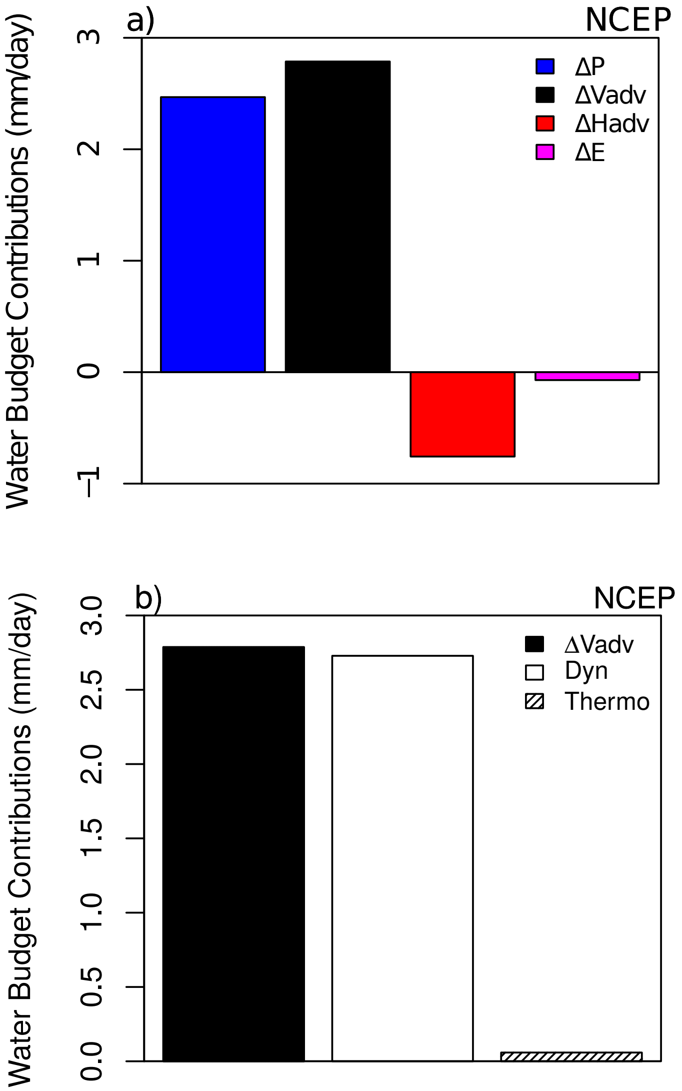

Figure 1: Monthly-mean anomalies for January 2014 of (a) precipitation, Vertical moisture advection, Horizontal moisture advection and Surface evaporation averaged over southern UK (50-52° N,6.5° W-0°) computed using NCEP, (b) dynamic and thermodynamic contributions to precipitation anomaly during January 2014 derived from Eq. (3) averaged over southern UK computed using NCEP. Anomalies are relative to 1981-2010 climatology.
